# Recent Progress in Laser Powder Bed Fusions Processes of Advanced High-Strength Steels

**DOI:** 10.3390/ma17194699

**Published:** 2024-09-25

**Authors:** Aleksandra Królicka, Julia Malawska

**Affiliations:** Department of Metal Forming, Welding and Metrology, Faculty of Mechanical Engineering, Wroclaw University of Science and Technology, 50-370 Wroclaw, Poland; 255110@student.pwr.edu.pl

**Keywords:** AHSSs, additive manufacturing, selective laser melting (SLM), laser powder bed fusion (LPBF), TRIP steels, TWIP steels, maraging steels, bainitic steels, low-density steels

## Abstract

This review is focused on the perspectives of the application of Advanced High Strength Steels (AHSSs) in the field of additive technologies directed at the laser powder bed fusion/selective laser melting processes. In principle, AHSSs require significant attention due to their promising mechanical properties for usage in the automotive industry towards reducing the weight of vehicles. Although additive manufacturing represents a promising perspective towards expanding the industrialization of AHSSs in a wider area of their applications, they have not been sufficiently investigated concerning their usage in LPBF/SLM processes. AM techniques enable the fabrication of complex machine parts, including those with a cellular structure, which can contribute to further reducing the weight of vehicles or structures. Maraging steels have recently attracted the attention of researchers, and today are a common grade of steel produced by LPBF techniques. The other group of AHSSs are high-Mn steels with an austenitic matrix characterized by the TRIP and TWIP effects. Less published research has been conducted on medium-Mn steels, which require additional intercritical annealing and preheating during printing. Moreover, the advanced bainitic steels and low-density, high-strength steels represent a new window for further research into the use of the LPBF processes for their fabrication.

## 1. Introduction

Considering the European and global directives relating to reducing CO_2_ emissions in the steel sector industry, developing new materials and adjusting advanced technology processes constitute crucial challenges to overcome. The purpose of current trends is focused on achieving high-strength and in-use properties of steels, which lead to the diminution of the structure’s weight and fuel consumption. Consequently, Advanced High-Strength Steels (AHSSs) are a material group of increasing interest in the scientific communities and the industry sector [1]. The scope of AHSSs mainly includes:

- dual-phase steels [2,3];
- multi-phase steels [4,5];
- medium-Mn steels [6,7];
- high-Mn steels [8,9];
- twinning-induced plasticity (TWIP) steels [10];
- transformation-induced plasticity (TRIP) steels [11];
- nanocrystalline bainitic steels [12,13,14];
- maraging steels [15].

Current research mainly focuses on controlling in-use properties and facilitating industrialization [16,17,18,19,20,21,22]. AHSSs constitute promising materials for use in the automotive industry considering the associated strong demands [23]. The vehicle parts made of AHSSs are prone to absorb more kinetic energy during the crash and are characterized by higher resistance to intrusion in comparison to low-strength steels [24]. The controlled contribution of the TRIP and TWIP effect constitutes a research direction focused on energy absorption capabilities, and high mechanical properties enable a reduction in vehicle weight. The significant in-use properties cover the main fields [24]: (i) weldability; (ii) coatability, primarily galvanic coatings; (iii) processability; (iv) cost; (v) recyclability. It was also indicated that the current trends for AHSSs constitute the light-weight alloy design strategy and improving the formability without the loss of mechanical properties level [25]. Although AHSSs are characterized by outstanding mechanical properties, their susceptibility to hydrogen embrittlement constitutes one of the particular challenges. For the majority of these steels, the local microstructure spots characterized by high gradients of local stress occur [1].

Over the last decade, there has been a significant increase in interest in additive manufacturing (AM) methods for producing demanding machine parts. Additive technologies provide prominent capabilities for production the of machine parts using various advanced engineering materials, and at the same time have a few crucial limitations. Moreover, the AM processes of steel constitute one of the relevant research trends [26].

Referring to current trends both in terms of novel material solutions and advanced technological processes, the scope of the current literature overview is focused on the laser powder bed fusion (LPBF) technology concerning AHSS. The LBPF is considered one of the most versatile additive manufacturing processes. It is used to produce metallic parts with complex geometries in a relatively short time. The increasing use of this method is associated with advantages such as the possibility of obtaining defect-free parts with good mechanical properties, probably due to the microstructure obtained during the process. Also, AM processes may constitute the solution for the limited formability and processability that occurred during processing in conventional manufacturing routes. Moreover, the use of, among others, cellular structures will enable further reduction of the machine parts, which is one of the advantages of AM techniques considering lightweight design. Despite these advantages, the LBPF process still has limitations due to the limited knowledge of the process and the influence of the parameters [27]. Moreover, the additive manufacturing trade beyond adjusting process parameters also includes, among other factors, the quality of the powder supplied to the process [28], which affects the repeatability of the process and the quality of produced parts. It should also be emphasized that the mechanical properties of the part of AHSSs are associated with the thermomechanical processes [29,30,31,32] that are not present during LPBF fabrication. This is also a significant limitation.

The LBPF process, also known as selective laser melting (SLM), is based on the use of a laser beam to melt particles of metal powder. A high-energy laser beam melts a specific contour layer by layer until a three-dimensional element is obtained. During the process, complex thermodynamic mechanisms take place, the energy absorbed by the powder particles generates a high temperature within them, and the nature of the process affects the high cooling rate. The interaction between the laser and the powder can be associated with problems such as balling, pore formation, and thermal cracking [27,33].

Based on a critical insight into the published investigations, future directions and prospects related to LBPF and AHSS processes were formulated. Moreover, the most crucial limitations and challenges were identified. The results of this literature analysis may constitute a basis for the design of future LPBF processes for wider grades of AHSSs and to optimize the process parameters aimed at controlling the microstructure and mechanical/in-use properties.

## 2. Insight into the Steels Manufactured by Laser Powder Bed Fusion

Laser powder bed fusion as one of the most common additive manufacturing processes for metal production has been successfully used to produce different types of steel. With the use of this technology, it has been reported to produce components with materials such as conventional steels, maraging steels, and AHSSs [34].

However, it has to be addressed that components produced with LPBF will be characterized by different microstructures than in conventional processes [26]. The focus of the current review is directed towards LPBF processes and AHSSs, and it may be noticed that this group of steels constitutes a relatively novel scope of materials considering addictive manufacturing methods.

### 2.1. Short Overview of the Steels Commonly Produced by LPBF Not Covering the AHSS Group

#### 2.1.1. Austenitic Stainless Steels (ASS)

Austenitic stainless steel produced by LPBF has been found to have excellent mechanical properties with an even better strength/ductility ratio, in comparison to conventionally produced components. The strength increase is sometimes attributed to microstructure obtained in the process, more specifically to the cellular or cellular–dendritic solidification structure. This specific microstructure is directly connected to the temperature gradients and rapid solidification rates inherent in the process [35,36]. AISI 316L is one of the most commonly used austenitic stainless steels in additive manufacturing, valued for its favorable mechanical properties and excellent corrosion resistance. The corrosion resistance of 3D-printed 316L is generally regarded as superior to that of forged material. It has been suggested that the cellular sub-structure formed during the laser powder bed fusion process enhances corrosion resistance. This is attributed to the segregation of molybdenum (Mo) and chromium (Cr) at the cell walls, creating a more corrosion-resistant barrier that helps limit the depth of corrosion attack [37]. The current state of additive manufacturing perspectives for ASS is formulated in an in-depth review developed by Haghdadi et al. [38].

#### 2.1.2. Duplex Stainless Steels (DSS)

Duplex stainless steels consist of approximately 50% ferrite and 50% austenite and combine beneficial corrosion resistance with mechanical properties and twice the strength of traditional austenitic stainless steels [39,40]. However, DSSs constitute the demanding material for the LBPF process as they still require post-processing such as solution annealing to control the austenite and ferrite content and morphology. The main problem with those steels produced by the LPBF process is that they demonstrate mainly ferritic microstructure, which is attributed to very high cooling rates. A high cooling rate inherent to LPBF processing favors the formation of a dominantly ferritic and N loss during the process [41]. To overcome this problem, dedicated heat treatment is used, and research indicates that the duplex microstructure may be successfully recovered. One of the commonly used grades of DSSs is 2205 with the composition of Fe-22Cr-5Ni-3Mo-1Mn (wt.%). For 2205 steel produced by LBPF, the solution annealing at 1100 °C for 1 h in an air atmosphere followed by water quenching is typically applied to control the ferrite and austenite content [40]. The current state of additive manufacturing perspectives for DSSs is demonstrated in an in-depth review developed by Zhang et al. [42].

#### 2.1.3. Oxide Dispersion Strengthened (ODS) Steels

Oxide Dispersion Strengthened steels represent a groundbreaking class of alloys, designed for industries demanding exceptional high-temperature resistance and mechanical strength. These materials are typically built on a ferritic/martensitic steel matrix, enriched with high levels of chromium (9–20%) to ensure corrosion resistance. Reinforcement comes from nanometer-sized dispersoids, which consist of titanium-containing yttrium-based oxides. In the nuclear industry, ODS ferritic alloys are particularly valuable as a potential alternative for radiation-resistant cladding and structural materials. Fine oxides, with their small size and high density, create numerous composite interfaces that help to eliminate point defects, preventing defect clustering and failure. This distinctive microstructure enhances their creep resistance and durability, making ODS steels the material of choice for applications in power plants, aerospace, and nuclear reactors [43,44]. The well-known way to fabricate ODS steel is mechanical alloying of Y_2_O_3_ which requires high-energy mechanical milling. Another way to produce ODS steel is the use of gas atomization reaction synthesis powders (GARS). The GARS-produced steel powders consist of a base ferritic steel matrix. As a result of the rapid solidification during atomization, stable Y-containing intermetallic (typically Y_2_Fe_17_) are formed along interdendritic regions or with Y that is retained in solution or at grain boundaries [45]. The current state of additive manufacturing perspectives for ODS steel is presented in an in-depth review developed by Wilms et al. [46].

#### 2.1.4. Precipitation-Hardened (PH) Stainless Steels

Precipitation-hardened steels are a class of high-strength stainless steels that achieve superior mechanical properties through the precipitation-hardening process. This process involves heating the steel to precipitate fine intermetallic compounds, which strengthen the material. PH steels offer an excellent combination of strength, corrosion resistance, and toughness, making them ideal for demanding applications in the aerospace, automotive, and chemical industries. They are commonly used in components like shafts, gears, and fasteners, where both high strength and resistance to harsh environments are essential. One of the most commonly used PH steel is 17-4 PH, this grade of steel is fully martensitic when it is fabricated through conventional methods such as forging, casting, or welding. PH steels are also commonly fabricated using AM [47,48,49,50]. However, when produced with the LBPF method, which involves extremely high-temperature gradients and rapid cooling rates, the microstructure is in a non-equilibrium state with metastable austenitic and BCC ferritic phases. PH steels produced by LPBF can be characterized by a wide range of phase compositions. Unlike conventionally manufactured PH 17-4 SS, LPBF-printed PH 17-4 SS can retain metastable austenite at room temperature [51]. It is found that 17-4 PH exhibits a complex, inhomogeneous microstructure with various amounts of δ ferrite, austenite, and martensite in the as-built condition. Research shows that even small variations of the chemical composition of used powders have a significant effect on the phase composition in the as-built samples [52]. The current state of additive manufacturing perspectives for PH stainless steel is included in reviews developed by Ko et al. [53] and Zadi-Maad et al. [54].

### 2.2. Advanced High-Strength Steels Produced by LPBF

#### 2.2.1. Medium- and High-Mn TWIP and TRIP Steels

Overall, the TWIP effect in steel occurs when the mechanical twins are formed during plastic deformation. When the steel is subjected to stress, instead of dislocation movement, twinning occurs, accommodating the deformation. TWIP steels typically have a medium or high Mn content. Medium-Mn steels refer to the 3–12 wt.% of Mn [55], while high-Mn steels are characterized by manganese content exceeding 13 wt.% [56]. The presence of martensitic transformation of retained austenite may be also the result of transformation-induced plasticity. Moreover, TWIP and TRIP steels benefit from the so-called “dynamic Hall–Petch effect”, a deformation-induced grain refinement leading to the evolution of new obstacles to dislocation motion [57]. TWIP steels are known for their excellent combination of high strength and ductility. This makes them perspective material for applications where the machine parts are subjected to absorbing large amounts of energy, such as in automotive crash components. Thus, TRIP steels also offer a beneficial balance between strength and ductility. The transformation to martensite absorbs energy, improving ductility while increasing strength.

The mechanical properties depend on the chemical composition of the steel and the volume fraction of retained austenite in its microstructure. The activation of the TRIP and TWIP deformation mechanisms is determined by factors such as volume fraction of structure constituents, grain size, and stacking fault energy (SFE) of the austenite [58], as well as the strain rate [59]. The current challenges of medium- and high-Mn steels are associated with the understanding of the physical metallurgy phenomena, twinning mechanisms, deformation mechanisms, texture evolution, fracture mechanisms, hydrogen embrittlement behavior, and fatigue performance [8,60,61].

**Figure 1:**
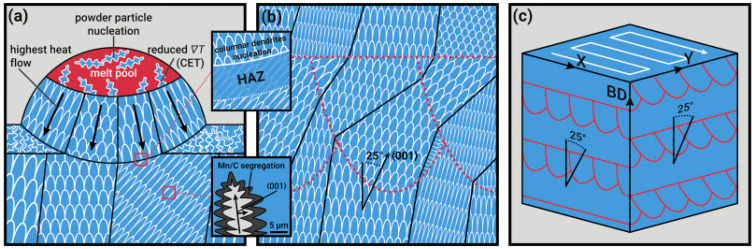
Microstructure evolution in AM manufactured high-Mn steel solidification. (**a**) The formation of columnar and equiaxed dendrites occurred during the deposition of a laser-melted track and heat-affected zone (HAZ). ∇T and CET indicate temperature gradient and columnar morphology into equiaxed morphology transition. (**b**) Origin of the texture caused by the solidification related to the 25° inclination of sustaining grains connected with the remelting process. (**c**) Texture originated from the route [64].

The high content of manganese may lead to difficulties with recrystallization processes, which typically leads to a depletion of manganese and may occur during high-temperature processes such as SLM. However, Niendorf et al. [62], first reported that the effective manufacturing of TWIP steels is achieved using the SLM technique. Steels produced with this process are characterized by mechanical properties similar to the conventionally processed material. Despite an anisotropic microstructure featuring elongated grains, preferred grain orientation, and process-induced imperfections such as inclusions and pores, the SLM-processed TWIP steel demonstrates high strength and high ductility even in a not-post-treated condition. The effective manufacturing of high-Mn TRIP and TWIP steels was also reported in other works [63,64,65,66,67]. The scheme of solidification processes during AM processes of high-Mn steels is presented in Figure 1. Haase et al. [63] investigated the high-Mn steel characterized by reduced chemical element segregation compared to conventionally manufactured alloys. The limited segregation constitutes a prospect to avoiding the additional post-treatment. Due to very fast cooling rates, the structure consisted of an austenite matrix with ε- and α’-martensite. Both, TRIP and TWIP effects were activated after LPBF processes. Authors suggest that the challenge related to LPBF processes of high-Mn steels is related to reduced formability. Ewald et al. [65] investigated the influence of Al content on the deformation mechanisms of LPBF-processed high-Mn steel. After the LPBF processes the TRIP and TWIP effects were observed, which contribution was related to the Al content. Moreover, Kohnen et al. [67] observed the tendency that increasing the Al content, a transition from austenite (FCC) to ferrite–austenite (BCC-FCC) occurs during solidification for 4 wt. % of Al. As a result, strong grain refinement and texture randomization were achieved. The presence of strong grain refinement and suppression of the contribution of the TRIP effect in the dual phase high-Mn steels (above 4 wt.%) plays a crucial role in enhancing the energy absorption capacity and ductile deformation behavior. Kies et al. [64] noticed that high-Mn steels subjected to LPBF processes are characterized by reduced segregation and suppressed grain boundary embrittlement. The authors proposed the fabrication of a lattice structure intended for energy-absorbing applications. Both TRIP and TWIP effects were revealed. Overall, high-Mn austenitic TRIP/TWIP steels are characterized by sufficient processability and demonstrate comparable mechanical properties compared to the hot-rolling processes without application of additional post-treatment. The overview of performed investigations is presented in Table 1.

**Figure 2:**
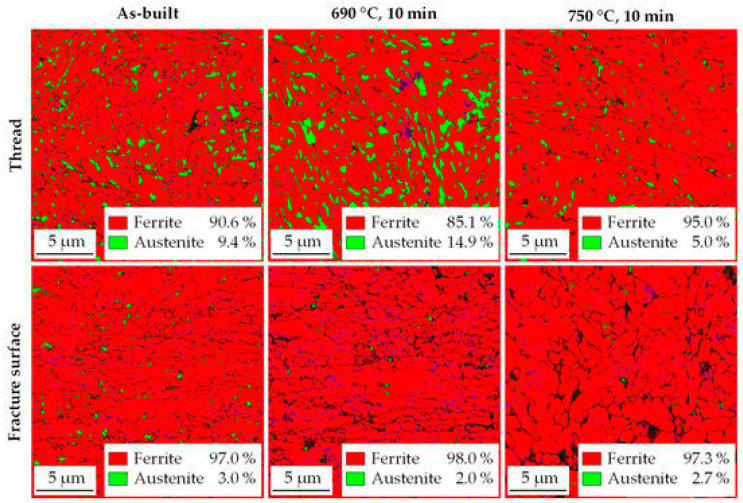
Phase distribution maps of medium-Mn steel manufactured using LPBF. Comparison of retained austenite content of as-built conditions and intercritical annealed processes at 690 °C and 750 °C. Area of the fracture surface indicates susceptibility to TRIP effect of retained austenite [69].

Pawlak et al. [68] and Heemann [69] reported investigations involving low-carbon, medium-Mn steel. In medium-Mn steels, a martensitic or martensitic–bainitic matrix with a controlled content of retained austenite typically occurs. In as-built conditions, the content of retained austenite is lower compared to conventional manufacturing routes. The post-treatment (intercritical annealing) significantly enhances the austenite susceptibility to the TRIP effect and increases the fraction of retained austenite compared to the as-built condition (Figure 2). It should be also noted that both papers applied preheating treatment at 200 °C to avoid cold cracking, and high relative densities were achieved (99.8–99.9%). The conducted research indicates that post-treatment (intercritical annealing) is necessary to enhance mechanical behavior up to the level of conventional manufacturing processes of medium-Mn steels.

#### 2.2.2. Maraging Steels

Maraging steels constitute a group of AHSSs characterized by ultra-high mechanical properties caused mainly by the precipitation strengthening of intermetallic compounds (among others Ni_3_Ti, Ni_3_Mo, Ni_3_ (Ti,Mo), Fe_2_Mo Laves phase, or B2-ordered NiAl) that are semi-coherent or coherent or with the low-carbon martensitic matrix [15,70,71]. Overall, maraging steel contains a low content of carbon (up to 0.03 wt.%) and a significant content of nickel (17–19 wt.%), together with lesser contents of cobalt (8–12%), molybdenum (3–5 wt.%), titanium (0.2–1.8 wt.%), and aluminum (0.1–0.15 wt.%). The alloying elements contribute to the formation of intermetallic phases during aging. The addition of Cr to the chemical composition results in the design of corrosion-resistant maraging steels [72]. The microstructure of maraging steels consists of a ductile martensitic matrix reinforced by the precipitation of nanometric intermetallic phases. The name “maraging” comes from the used heat treatment. These steels are subjected to heat treatment—hardening and then aging at a temperature of 450–510 °C—which leads to a significant increase in hardness and strength properties as a result of intermetallic strengthening. The current challenges associated with the development and industrialization of maraging steel include stress corrosion cracking of welded joints, fatigue life, and deformation behaviors [73,74,75].

**Figure 3:**
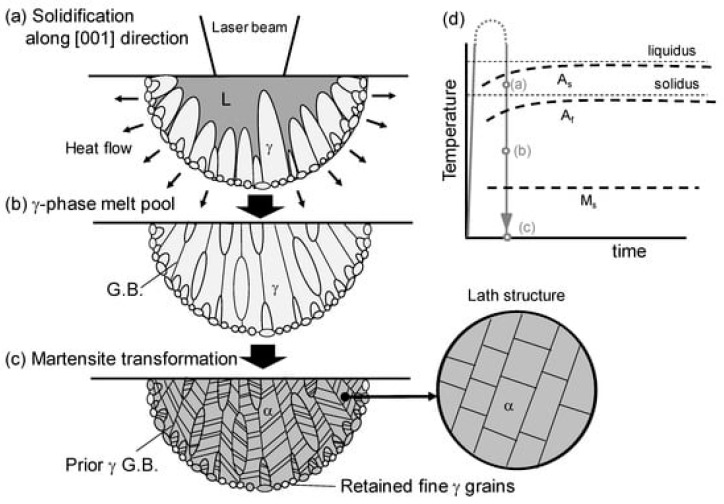
(**a**–**c**) Scheme of microstructure formation of maraging steel during SLM/LPBF processes. (**d**) Thermal history of the sample related to local laser heating [82]. γ—austenite; G.B.—grain boundary; α—ferrite; M_s_—martensite start temperature; A_s_—solidification temperature.

Maraging steels are commonly manufactured using additive manufacturing, especially LPBF processes. In recent years, a few in-depth review papers have been published on the current developments in AM and manufacturing of maraging steels [76,77,78,79]. For this reason, the scope of this literature review presents the most crucial assumptions and findings regarding maraging steel and LPBF processes focused on papers published during the last few years. It is stated that the AM methods and additional heat treatment (aging processes) enable the manufacturing of the maraging steels characterized by comparable strength properties and hardness properties compared to the conventionally fabricated parts [76,80]. The differences in the ductility and strength properties of maraging steels are related to the presence of retained and reversed austenite caused by the chemical inhomogeneity related to the micro-segregation upon solidification [81] (visible also in Figure 3). It is also revealed that after AM processes the anisotropy of mechanical properties occurs related to the build orientation [77]. For the final structure and quality of LPBF-fabricated maraging steels, the process parameters play a crucial role (laser power, hatch distance, laser scanning velocity, build direction, layer thickness). The influence of process parameters on the structure, defects, and properties of maraging steels is presented in a review developed by B.S Rao and T.B. Rao [79]. One of the advantages of producing maraging steels by AM processes such as LBPF is the intrinsic rapid solidification rate during the process, which may facilitate the austenite to martensitic phase transformation and reduce the post-heat treatment time compared to the conventional manufacturing process [81].

Based on recent findings, a summary is presented regarding the fabrication of maraging steel using LPBF/SLM processes (also in Table 2). It is reported that one of the most commonly used maraging steels in LPBF processes is the M300 grade. This grade is characterized by the capability to produce parts without cracks and minimum porosity, and after postprocessing may demonstrate an expected strength and fracture toughness combination. Wei et al. [83] revealed that approximately 9.2% of retained austenite in the as-build sample is preserved. Takata et al. [82] noticed that fine retained austenite with a <001> orientation along the build direction is mainly formed at the melt pool boundaries. After complete heat treatment (quenching + aging; and solutionizing + austenitizing) it is possible to obtain fully martensitic microstructures [83,84] Moreover, during the direct aging process, austenite reversion occurs and may lead to an increase in the overall austenite fraction [83,85].

Król et al. [86] revealed the presence of two solid-state reactions during the heating of SLM-manufactured maraging samples:

- precipitation of intermetallic phases;
- reversion of martensite into austenite.

Based on the Kissinger equation, the authors indicated the activation energy for each reaction (precipitation of intermetallic phases 301 kJ mol^−1^, the martensite to austenite reversion 478 kJ mol^−1^). Moreover, Eres-Castellanos et al. [87] revealed that during reheating related to the subsequent deposition, grains subjected to re-austenitization keep the same crystallographic orientation as the surrounding retained austenite. Santana et al. [88] demonstrated that no crucial influence of the used printing parameters on the microstructure evolution occurred during aging. Furthermore, it was noticed that the hardness of aged maraging steel is comparable to that obtained by conventional manufacturing routes. Kannan et al. [89] observed the direct aging processes for Ti-free maraging steel manufactured using LPBF for various aging times. The authors indicated that aging at a lower temperature (or shorter time) led to reduced strain hardening caused by a lower content of reverted austenite. On the other hand, aging at a higher temperature led to extensive recrystallization of martensite, precipitation coarsening, and significant austenite reversion, which caused the softening of the fabricated samples. Piekło et al. [90] developed the relation between aging temperature and fatigue life. From the perspective of corrosion resistance, Zhao et al. [91] demonstrated that SLM-fabricated 18Ni300 maraging steel was negatively correlated to the size of pores formed during printing. A.G. Demir and B. Previtali [92] attempted to use the preheating of the 18Ni300 maraging steel (at 170 °C), aiming at reducing the porosity. However, the preheating was not significantly effective in terms of enhancing the density.

**Table 2:** Overview of selected maraging steels subjected to LPBF processes.

	Powder	Parameters	Comments	Ref.
X3NiCoMoTi18-9-5 steel (in wt %)	Gas atomized, particle size: 15 to 45 μm	Energy density: 60–70 J/mm^3^, Laser powder: 300 W, Scanning speed: 1 cm/s, Layer thickness: 50 μm, Argon gas atmosphere	In as-built state: elongated grains with nano-scale cellular dendritic structure. This microstructure transformed into a martensitic lath microstructure after heat treatment. The retained austenite completely transformed into martensite after solution annealing heat treatment. The aging process produced reverted austenite.	[85]
M789 (0.02C-12.2Cr-10Ni-1Mo0.06Al-1Ti-Fe)	Gas atomized, Particle size: 15–45 μm	Energy density: 75–85 J/mm^3^ Argon gas atmosphere	In as-built structures: melt pool boundaries with nano-scale columnar dendritic structures were present. The elongated grains transformed into martensitic needle-like structures after annealing and aging treatments.	[93]
18Ni-350 (18.44Ni-11.91Co-4.88Mo-1.45Ti-0.12Al-0.01C)	Gas-atomization under nitrogen environment, average particle size: 30 μm	Laser power: 175 W, Hatch spacing: 100 μm, Scan speed: 300 mm/s, Layer thickness: 30 μm, Scan rotation: 67°, Energy density: 194.4 J/mm^3^, Relative density: 99.6–99.8 %	After direct aging, austenite reversion occurred, increasing the overall austenite fraction (21.8%). Austenitizing–aging, and solutionizing–austenitizing–aging treatments eliminated the austenite and a fully martensitic microstructure was achieved. A high density of dislocations was found in the martensitic laths in all the conditions examined.	[83]
18Ni-300 0.025Mn-0.01Cr-4.5Mo-18Ni-0.07Al-9.2Co-0.91Ti	Atomized in an inert argon medium using electrode induction melting gas atomization, average particle size: 40 um	Laser power: 300 W, Focus diameter: 70 μm, Scan speed: 1300 mm/s, Hatch space: 70 μm, Layer thickness: 30 μm, Rotation angle: 33°	Absence of nano-precipitate in the as-built state, while precipitations of Ni-Ti-based and Mo-based precipitates were confirmed in both the aged and solution-aged states.	[94]
18Ni-300	Gas atomized, particle size: 15 to 53 μm	Laser power: 285 W, Scanning speed: 960 mm/s, Hatching space: 110 μm, Layer thickness: 40 μm, Scanning strategy: Orthogonal, Relative density: >99.5%	Significant anisotropies in the thermal expansion and CTE were observed between the OX (parallel) direction and OZ (perpendicular) direction in the LPBFed samples. The solution-treated and solution+ageing-treated samples showed differences in the coefficient of thermal expansion CTE compared to direct aging-treated samples.	[95]
18Ni-300	Gas atomized, average particle size: 88.8 μm	Laser power: 2000 W, Scanning speed: 1000–2000 mm/s, Hatch distance: 0.06–0.1 mm, Layer thickness: 0.1–0.2 mm, Hatch angle: 90°, Energy density: 50–180 J/mm^3^, Build rate: 6–40 mm^3^/s	High power (2000 W) contributed to typical HP-LPBF dendritic microstructure inside columnar prior-austenite grains (PAGs) growing along the building direction. The original cellular and columnar dendrites were replaced by parallel lath martensite and nanoprecipitates could be observed in the sample with solution-aging treatment.	[96]

Based on the literature overview, the following summary was formulated:

- Maraging steels constitute a steel group that may be effectively fabricated using LPBF/SLM processes after adjusting the process parameters to achieve high-density samples and reduced fraction of defects;
- The mechanical properties of LPBF-fabricated maraging steels are generally comparable to the conventional manufacturing routes;
- The LPBF-fabricated maraging steels to achieve expected mechanical properties are subjected to heat treatment (quenching + aging; solutionizing+austenitizing) or direct aging;
- During aging, the intermetallic precipitation growth is similar to the conventional manufacturing routes;
- During aging, the martensite is prone to transformation into reverted austenite;
- In the as-built conditions, the retained austenite is preserved. The retained austenite is formed especially at the melting pool boundaries due to occurred micro-segregation of local chemical composition.

#### 2.2.3. Bainitic Steels

Over three decades, ultra-fine bainitic steels have attracted scientists and the industry sector due to their beneficial combination of mechanical properties in comparison to conventional martensitic steels [97,98,99]. Obtaining a bainitic structure with ultra-fine or even nanoscale dimension is related to the solid-state reaction at low temperatures and through dedicated alloy design high silicon steels [14]. The structure of ultra-fine bainitic steels contained carbon-super saturated bainitic ferrite and stable retained austenite [100]. The higher content Si (typically above 1.5 wt.%) leads to incomplete transformation and retained austenite stabilization at room temperature [101]. A similar role to Si is associated with Al considering the suppression of cementite precipitation from austenite [102]. The bainitic steels with increased Al and Si content due to limited carbide precipitations are called carbide-free [103]. The current challenges for the development of advanced bainitic steels cover, among other, the strategy for reducing carbon content, fatigue and in-service performance, the thermal stability of the metastable structure, deformation behavior, and the thermomechanical processes [104,105,106,107,108].

Despite extensive research covering the development of advanced bainitic steels, the current challenge to overcome is connected to extending industrial applications [17]. AM processes in the manufacturing of ultra-fine bainitic steels open a new window related to their potential applications. The majority of the literature [109,110,111,112,113] indicate that the most common AM process for producing bainitic structures is Wire and Arc Additive Manufacturing (WAAM).

**Figure 4:**
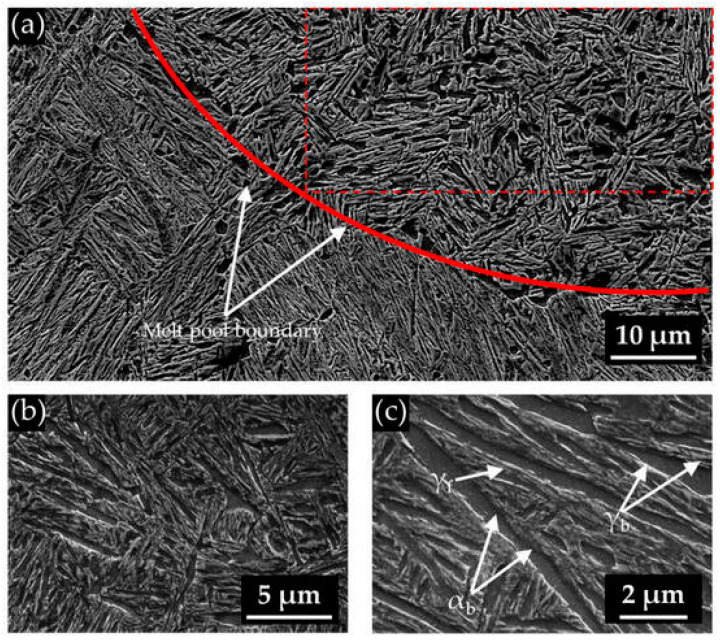
(**a**–**c**) Bainitic microstructure at prior melt pool boundary. γ_f_—austenite films, γ_b_—austenite blocks, α_b_—bainitic ferrite [115].

Prior to publication of this paper, only a few published works have attempted to produce bainitic steels using LPBF processes (Table 3). Bartels et al. [114] investigated low-carbon bainitic steel subjected to LPBF processes. The minor content of retained austenite was detected in as-built conditions, while post-treatment led to the reduction of their content. The bainite morphology was different in the fusion zone and heat-affected zone (upper and lower bainite with cementite precipitations). Franceschi et al. [115] investigated carbide-free bainitic steel subjected to the LPBF process. The samples after LPBF were characterized by a refined carbide-free bainitic structure due to the formation of smaller prior austenite grains in comparison to conventional manufacturing processes. Moreover, the samples subjected to LPBF demonstrated a faster bainitic transformation kinetic caused by the finer prior austenite grain size. The inhomogeneity of structure constituents was noticed within the single layer following the layer-by-layer manufacturing process. After the deposition on a novel layer, the austenitized layer will transform into martensite, and further layers will be subjected to tempering processes. The post-treatment involved a complete heat treatment route for ultra-fine bainitic steels. However, the structure after post-treatment was characterized by various bainite morphologies and bimodal distribution of bainitic ferrite plate thickness in the fusion zone, core, and heat-affected zone (Figure 4).

The lack of literature related to the production of LPBF ultra fine bainitic steels is directly related to its limitations:

- The ultra-fine bainitic steels are characterized by medium or high carbon content. The bainitic structure is achieved during prolonged isothermal heat treatment. Thus, during fast cooling after the process, the cold cracks related to martensite transformation are prone to be formed, similar to welding processes [116]. The solution for avoiding the cold crack is the usage of heated support with the temperature above martensite start (M_s_) temperature.
- The LPBF is connected with formation layer-by-layer. The metastable bainitic structure together with the limited thermal stability of retained austenite tend to the decomposition process [106] and the deposition of subsequent layers. This process is similar to the tempering process.
- It seems that LPBF-fabricated bainitic steel should be subjected to a complete post-treatment process similar to conventional heat treatment (quenching and isothermal heat treatment). However, the processing of bainitic steels with lower carbon content and faster bainitic transformation may be produced using heated support at the temperature of bainitic transformation during the whole LPBF process. However, due to the segregation of chemical elements, the distribution of retained austenite may be not homogeneous in the fusion zone and heat-affected zone [115].
- The future direction of investigations of AM processes of bainitic steels is also associated with the fabrication capabilities of secondary hardened bainitic steels [117,118,119], continuous cooling bainitic steels [120,121,122], and intermetallic strengthened bainitic steels [123,124,125].

The experimental studies of developing the optimized LPBF technology for ultra-fine bainitic steels constitute a research gap and require further research aimed at controlling the structure morphology and economical aspects of manufacturing. It cannot be ruled out that there is a necessity to develop a new design alloy strategy oriented towards AM processes to overcome the most crucial difficulties.

#### 2.2.4. Novel Concepts of Advanced Steels—Low-Density High-Strength Steels

**Figure 5:**
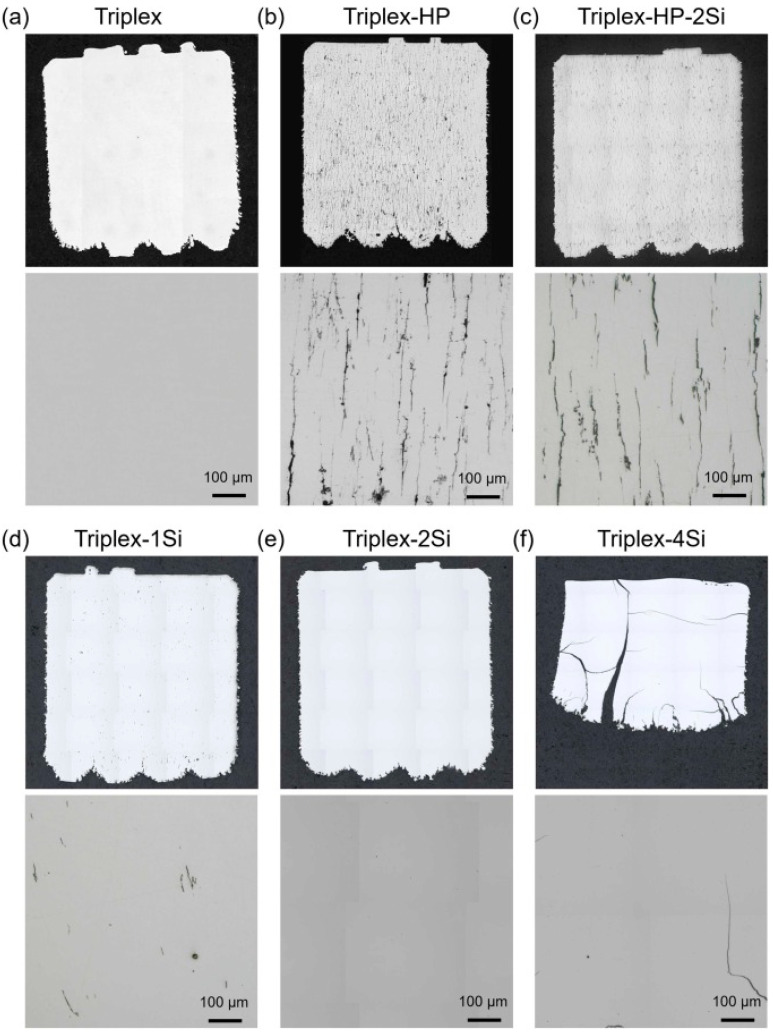
(**a**–**f**) Overview of hot cracking presence in six various TRIPLEX grades (Triplex, Triplex-HP, Triplex-HP-2Si, Triplex-1Si, Triplex-2Si, Triplex-4Si) produced by LPBF process with parameters: 200 W, 700 mm/s, and VED of 130 J/mm^3^ [137].

The necessity for reducing the weight of steel for structural purposes has been growing, especially considering the transportation and automotive sectors [126]. Thus, a novel class of high-strength steels called low-density or lightweight steels was developed [127]. The current challenges for low-density steels include optimization of heat treatment, reducing the cost of alloys, and formability [128]. The promising grade of low-density steels is TRIPLEX steels (Fe-Al-Mn-C alloy system) characterized by a microstructure consisting of an austenite matrix and finely dispersed nanoscale κ-carbides [129,130,131]. Overall, the industrial applications of high-Mn TRIPLEX steels are limited due to the inherent difficulties related to conventional manufacturing processes, among others: (i) the necessity of performing energy-intensive post-treatment caused by strong segregation [132]; (ii) limited machinability [133]; (iii) reduced high-temperature formability [134]; and (iv) generation of reactive slag [135]. It seems that these challenges may be solved by using powder additive manufacturing processes aimed at directly fabricating components considering minor post-processing. Seede et al. [136] were the first to introduce the LPBF to produce steel with reduced density (Fe-30Mn-9Al-1Si-0.5Mo-9C wt.%). Promising tensile properties were achieved (ultimate tensile strength up to 1.3 GPa, while elongation was approximately 36% considering the build direction). However, after the LPBF processes, the presence of solidification micro-cracks in the whole cross-sectional area related to the hot cracking was detected. The microstructure constituted of austenite with cellular solidification morphology. Moreover, the strong micro-segregation of Si and Mn was revealed in the cellular–dendritic structures, as well as macro-segregation at the melt pool boundaries. On the other hand, Sanchez-Poncela et al. [137] were the first to manufacture TRIPLEX steel using LPBF technology. Based on the performed investigations, the hot cracking HC is prone to be formed when the alloy composition is not tailored. The hot cracking occurs as vertical cracks along the as-building direction and further may propagate across the whole cross-section of produced parts (Figure 5). The authors suggest that the presence of hot cracks in TRIPLEX steels may be predicted using hot cracking coefficient (HCS) criteria determined based on the solidification curve determined by the CALPHAD method. Particular attention was paid to the P and S, which segregate into the liquid during solidification by forming thin regions enriched with these chemical elements, which constitute a potential risk for hot cracking. Despite that austenite is the matrix of TRIPLEX steels, the ferrite formation at the dendrite boundaries was noticed for alloys characterized by higher Si content. Based on performed tests, the authors stated that AM processes constitute a reliable alternative to conventional metallurgical processes aimed at achieving thin, small, or complex shape components produced from low-density TRIPLEX steels.

However, the scientific field of low-density steels and AM processes still constitutes a large research gap and requires further investigations focused on eliminating cracks, micro- and macro-segregation, controlling the morphology of the microstructure and carbide precipitation, and tailoring the structure–property relationship. A crucial role is also played by process parameters and the development of effective post-treatment processes aimed at enhancing the mechanical properties and enabling the producing larger parts.

## 3. Conclusions

Based on the literature overview, a review of AHSSs produced by additive manufacturing methods was performed, focusing on laser powder bed fusion/selective laser melting processes. Although maraging steels are a common group of steels produced by AM techniques, other AHSSs still constitute a large research field where there are more questions than answers. AM techniques open a new window of perspectives towards the industrialization of steel grades with excellent mechanical and in-use properties, which are promising for reducing the weight of structures and designing steels with low environmental impact. The most relevant findings from the literature review include:

- Maraging steels: Adjusting process parameters enabled the fabrication of the parts with high density and a limited fraction of defects. After heat treatment or direct aging, strengthening by intermetallic phases is achieved similarly to conventional manufacturing processes. After the LBPF processes, the structure retains austenite located mainly at the melting pool boundaries, which is related to the micro-segregation of the chemical composition. During aging, the transformation of the martensite into reverted austenite occurs.
- High-Mn steels with TWIP/TRIP effect: Mechanical properties after LPBF are comparable to the conventional processing. The fully austenitic matrix is achieved. Additional post-printing heat treatment is not necessary to introduce TWIP and TRIP effects. The design strategy to reduce the segregation of chemical elements is favorable for AM processes. Both ε- and α´-martensite are formed during deformation. The contribution of TRIP and TWIP effects is related to the Al content.
- Medium-Mn steels with TRIP effect: A martensitic or martensitic–bainitic matrix with a controlled content of retained austenite is formed during the LPBF. Martensite is also subjected to the tempering process during printing. In as-built conditions, the content of retained austenite is lower compared to conventional manufacturing routes. The post-process heat treatment (intercritical annealing) significantly enhances the austenite susceptibility to the TRIP effect and increases the fraction of retained austenite compared to the as-built condition. Preheating at 200 °C is used to avoid cold cracking. The conducted research indicates that post-process heat treatment (intercritical annealing) is necessary to enhance mechanical behavior up to the level of conventional manufacturing processes of medium-Mn steels.
- Bainitic steels: They still constitute a research gap. After the LPBF process, a complete heat treatment allows for the formation of the expected carbide-free bainitic microstructure, which is, however, still characterized by heterogeneity. In terms of bainitic steels, a new chemical composition design strategy is required to achieve a bainitic structure during printing characterized by a combination of strength and ductility. Process parameters also require further investigation to prevent cold and hot cracking.
- Low-Density, High-Strength Steels: Although the low-density still attracts huge attention of researchers, the AM processes constitute a large research gap and require further investigations focused on eliminating cracks, micro- and macro-segregation, controlling the morphology of the microstructure and carbide precipitation (especially Kappa carbide), and tailoring the structure–property relationship. A crucial role is also played by process parameters and the development of effective post-treatment processes aimed at enhancing the mechanical properties and enabling the producing larger parts. Only TRIPLEX steel and Fe-30Mn-9Al-1Si-0.5Mo-9C were subjected to LPBF processes, which is a promising premise for further research.

Based on the analysis of the published papers on the LPBF/SLM technique and AHSS fabrication, this issue constitutes a significant research challenge. Extensive research into the manufacturing of maraging steel confirms the effectiveness of LPBF techniques in achieving mechanical properties comparable to conventional manufacturing routes. However, several phenomena in this area require further explanation and understanding (e.g., chemical composition segregation, the relationship between the retained austenite reversion and intermetallic formation, and the relationship between the texture and process parameters). When it comes to high- and medium-Mn, bainitic, or high-strength, low-density steels, there are still more questions than answers. It is difficult to state unequivocally whether LPBF techniques will be truly effective in fabricating these steel grades, which requires further experimental research. However, research on high-Mn and medium-Mn steels is prospective in these terms. On the other hand, bainitic steels and high-strength, low-density steels constitute a research gap, as confirmed by the first published works (published in 2024: TRIPLEX steel [137] and carbide-free bainitic steel [115]). Therefore, AHSSs represent an open window for future research and constitute currently a “hot” research topic.

## Figures and Tables

**Table 1:** Overview of medium- and high-Mn steels subjected to LPBF processes.

	Powder	Parameters	Comments	Ref.
Fe-0.15C-0.46Si-7.21Mn-1.81Mn (wt.%) Medium-Mn steel	Argon atomization, particle size: 20–65 μm	Layer thickness: 30–60 μm, Laser power: 250–350 W, Scanning speed: 700–1000 mm/s, Linear energy: 0.25–0.50 J/mm, Preheatment: 200 °C, Relative density: 99.76–99.87%	Intercritical annealing at 670 °C (0.5–6 h). The as-built microstructure: lath martensite and retained austenite (~8%). Microstructure after intercritical annealing: tempered martensite and retained austenite (~40%). Increased strength properties, elongation, and impact toughness. TRIP effect.	[68]
Fe-0.23C-3.93Mn-2.01Al-0.51Si (wt.%) Medium-Mn steel	Atomized using a Close-Coupled-Atomizer, particle size: 20–63 µm	Layer thickness: 60 μm, Laser power: 300 W, Scanning speed: 500 mm/s, Preheatment: 200 °C, Energy input: 111.1 J/mm^3^, Relative density: 99.9%	Intercritical annealing at 630–770 °C (3, 10 and 60 min). The as-built microstructure: bainitic–martensitic matrix and retained austenite. Microstructure after intercritical annealing: martensite/bainite, M/A constituents, and retained austenite. After post-treatment mechanical behavior was comparable to conventional production routes. TRIP effect.	[69]
Fe-0.60C-22.36Mn-0.25V-0.2Cr-0.25Si (wt.%) High-Mn steel	Spray aeration in an argon inert gas, particle size: 40 μm	Laser power: Up to 400 W	An anisotropic structure covering elongated grains, texture, and process-related defects (inclusions and pores). Mechanical properties after LPBF are comparable to the conventional processing. Dynamic Hall–Petch effect was revealed. TWIP effect.	[62]
Fe-0.27C-20.15Mn-0.05Si-0.01Al (wt.%) High-Mn steel	Electrode induction melting gas atomization with argon, particle size: below 45 μm	Scan line spacing: 100 μm, Scanning speed: 571 mm/s, Relative density: 99.9%	Due to the reduced chemical elements segregation compared to strip-cast material the additional post-treatment may be avoided. Revealed crystallographic texture related to the solidification affected the mechanical behavior. The austenite matrix contained ε- and α′-martensite. TRIP and TWIP effect.	[63]
Fe-0.33C-21.9Mn-0.01Al-0.03Si (wt.%) High-Mn steel	Gas-atomized, particle size: 15–50 μm	Layer thickness: 30 μm, Laser power: 120 W, Scanning speed: 700 mm/s	SLM lattice structure manufacturing. Reduced segregation after LPBF processes. TRIP and TWIP effects. Formation of ε- and α′-martensite during deformation.	[64]
Fe-(21-22)Mn-(0-5)Al-0.3C (wt.%) High-Mn steel	-	Layer thickness: 30 μm, Laser power: 90–120 W, Scanning speed: 200–700 mm/s, Relative density: 99.85–99.95%	TRIP and TWIP effects were revealed after the LPBF process. The Al content influenced the contribution of TRIP and TWIP effects.	[65]
X30Mn21 steel and controlled content of aluminum powder (0.03–5.39 wt.%) High-Mn steel	Argon gas atomized, particle size: 10–45 μm	Layer thickness: 30 μm, Laser power: 120 W, Scanning speed: 750 mm/s, Relative density: 99.9%	TRIP and TWIP effects were revealed after the LPBF process. Fully austenite matrix below 4 wt.% of Al. Above 4 wt.% of Al the dual phase structure occurs (ferrite and austenite).	[67]

**Table 3:** Overview of bainitic steels subjected to LPBF processes.

	Powder	Parameters	Comments	Ref.
Fe-0.22C-0.7Si-1.2Mn-1.0Cr-0.9Mo-(wt.%) Low-carbon bainite	Particle size: 15–45 μm	Layer thickness: 60 μm, Laser power: 225–275 W, Scanning speed: 550–850 mm/s, Relative density: above 99.7%	Post-treatment: tempering (150–600 °C), quenching, and tempering. Various bainite morphologies in the fusion zone and heat-affected zone. Post-treatment reduced the retained austenite.	[114]
Fe-0.4C-3.2Si-2.6Mn-0.1Al Medium-carbon carbide-free bainite	Argon atomization particle size: 15–53 µm	Layer thickness: 60 μm, Laser power: 170–295 W, Scanning speed: 500–750 mm/s, Energy density: 22–89 J/mm^3^, Pore density: from 2.15%	Post-treatment: austenitization at 900 °C and isothermal annealing at 325 °C for 3 h. A refined carbide-free bainitic structure due to the smaller prior austenite grains formed during the LPBF process. Non-homogeneous bainite morphology. The cracks were observed.	[115]
